# Differential gene expression indicates modulated responses to chronic and intermittent hypoxia in corallivorous fireworms (*Hermodice carunculata*)

**DOI:** 10.1038/s41598-021-90540-9

**Published:** 2021-05-27

**Authors:** C. J. Grimes, L. H. Petersen, A. Schulze

**Affiliations:** Texas A&M University at Galveston, TX, 200 Seawolf Parkway, Galveston, TX 77554 USA

**Keywords:** Ecology, Evolutionary ecology

## Abstract

Climate models predict an increase in extent, frequency, and duration of marine hypoxia events in the twenty first century. A better understanding of organismal responses to hypoxia in individual species is a crucial step for predicting ecosystem responses. We experimentally subjected a common invertebrate, the bearded fireworm (*Hermodice carunculata*) to two levels of chronic hypoxia and, in a separate experiment, to intermittent hypoxia. We found components of the conserved hypoxia-inducible factor (HIF) pathway and show a modulated response to hypoxia depending on the severity of hypoxic stress: under mild hypoxia, only the HIF-1α subunit is upregulated, while expression of the other subunit, aryl hydrocarbon nuclear translator, only increases significantly at more severe hypoxia levels. The chronic trials revealed down-regulation of genes related to cell adhesion, transport, development and heme-binding, and up-regulation of genes related to glycolysis, oxygen binding, cell differentiation, digestive and reproductive function. The intermittent hypoxia trials revealed an upregulation of heme transporter activity during hypoxia, and our time series analysis characterized nine clusters of genes with similar expression patterns. Our findings suggest that *H. carunculata* is likely to tolerate, and be resilient to, predicted future hypoxia conditions.

## Introduction

Low dissolved oxygen (DO), or hypoxia, events are increasing in area, frequency and duration as the oceans continue to warm and anthropogenic nutrient runoff to coastal ecosystems continues^1–3^. Hypoxia has recently come into focus as a stressor in coral reef ecosystems^1,4–6^ which are already severely decimated by coral disease, sedimentation, reduced herbivory, ocean acidification and rising temperatures^7,8^. Understanding how marine species respond to environmental stressors, including hypoxia, is crucial for predicting ecosystem-level responses (e.g.^9–11^). When marine hypoxia events occur, size and mobility of animals play important roles in their response^12^. Large mobile organisms may simply move towards normoxia (> 5 mg O_2_ L^−1^) when DO is lowered (e.g.^10,13–15^), while small sediment-dwelling organisms may crawl to the surface (e.g.^16,17^). Sessile or slow-moving animals have limited options to endure or escape hypoxia^18–21^ and their survival depends on physiological, and sometimes morphological responses, to cope with low oxygen levels.

Hypoxia is often defined as dissolved oxygen (DO) levels below 2 ml O_2_ L^−1^^22,23^ but this threshold is arbitrary, as different organisms exhibit different sensitivities to diminishing O_2_ and even slightly reduced oxygen levels can negatively impact some organisms^24^. The term “hypoxic” is context-specific and can either refer to the level at which an organism responds to low oxygen conditions or to the environment in which the organism resides. For the purpose of this study, we characterize DO concentrations of 4.5 mg O_2_ L^−1^ as Mild hypoxia^25,26^, 2.5 mg O_2_ L^−1^ as Moderate hypoxia, and 1 mg O_2_ L^−1^ as Severe hypoxia. Our previous studies indicate that previous exposure to the Moderate hypoxia increases *H. carunculata*’s metabolic rate in the long-term^27^.

**Figure 1:**
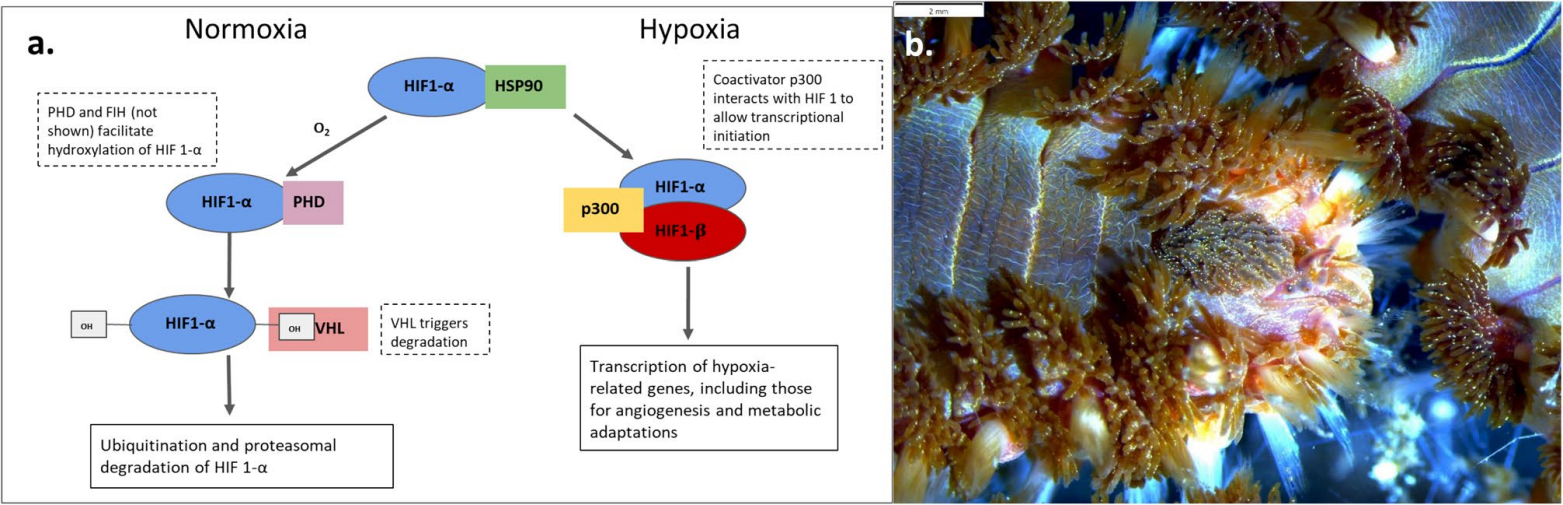
(**a**) HIF-1 pathway under normoxic (left) and hypoxic conditions (right). HSP90 is a heat shock protein that stabilizes HIF1-α until (under normoxic conditions) O2 facilitates the hydroxylation of HIF-1α at two proline residues with proline hydroxylase (PHD) and one asparagine residue with factor inhibiting HIF-1α hydroxylation (FIH) (created and modified from Liu and Semenza 2007). (**b**) Anterior end of the bearded fireworm (*Hermodice carunculata*) showing the primary tissues for oxygen-uptake which are the red branching branchia (similar to gills or lungs). Photo by CJ Grimes.

Throughout metazoans, hypoxia responses are triggered by the conserved hypoxia inducible factor (HIF) pathway^28,29^ which activates downstream processes (e.g. blood vessel formation, metabolic depression, etc.)^21,30–32^. The HIF transcription factor consists of two constitutively expressed subunits known as HIF-1α and aryl hydrocarbon nuclear translator (ARNT) or HIF-1β (Fig. 1a). Under normal oxygen conditions, HIF-1α is unstable and a functional transcription factor cannot form. HIF-1α is hydroxylated at one of two proline residues by the oxygen-dependent enzymes proline hydroxylase (PHD) or factor inhibiting HIF-1α hydroxylation (FIH)^31^. The Van-Hippel-Landau protein (VHL) binds to the hydroxylation site and leads to the addition of a poly-ubiquitin tail to HIF-1α, marking it for proteasomal degradation. Under hypoxic conditions, HIF-1α is not hydroxylated and forms a heterodimer with the ARNT and relocates from the cytoplasm to the nucleus as a functional transcription factor to trigger downstream responses^31^.

*Hermodice carunculata* (Pallas, 1766) is an amphinomid annelid (Fig. 1b) which is abundant and widespread throughout the tropical and subtropical Atlantic. It exhibits high tolerance to environmental extremes (reviewed in^33^) and is usually regarded as a nuisance species in coral reefs, as it feeds on live corals, leaving distinctive feeding scars^34,35^ and hindering coral reef restoration efforts^36,37^. Although the bearded fireworm is motile, its slow speed and limited movement range are likely to impede its escape from a large-area hypoxic event. In captivity, this amphinomid climbs toward the surface of the water when DO is lowered, either by crawling up the wall of aquaria or onto rock surfaces^38,39^. Recently, Yáñez-Rivera and Salazar-Vallejo^34^ proposed the existence of two species: *H. carunculata* in the Western Atlantic and *H. nigrolineata* Iin the Eastern Atlantic, and the distinction between the two was based primarily on the number of branchial filaments^40^. However, based on molecular evidence^41^ and phenotypic plasticity of branchiae in response to DO conditions^38,39^, *H. nigrolineata* is now considered a junior synonym to *H. carunculata*.

Among marine invertebrates, gene expression in response to hypoxia has been particularly well studied in crustaceans (e.g.^42–45^) and bivalves (e.g.^46–49^) due to their ecological and economic importance. In the Pacific oyster, *Crassostrea gigas* (Thunberg 1793), hypoxia exposure led to increased stress response, glutamine synthetase and metallothionein concentrations^50^, and an overexpression of genes related to respiration, metabolism, and immune system^51^. To date, few gene expression studies in response to hypoxia have been performed on annelids. Recently, Ogino and Toyohara^17^ showed that the sediment-dwelling *Capitella teleta* expresses TRP1A, a hypoxia-sensor which identifies the severity of hypoxic conditions to determine whether or not to trigger a behavioral response. The hydrothermal vent-inhabiting *Paralvinella* sp. up-regulates extracellular hemoglobin but down-regulates intracellular hemoglobin under hypoxia^52,53^. An increased production of extracellular hemoglobin may provide a more efficient way to bind and transport the limited oxygen than intracellular hemoglobin which appears to be more important for longer-term oxygen storage.

Intermittent hypoxia occurs in common habitats of *H. carunculata*, such as tide pools^54,55^ and on coral reefs^1,56^, while chronic hypoxia is less common in their typical habitat due to algae and coral symbiont primary productivity during the day^4^. However, based on climate change models, chronic hypoxic events are more likely in the future^1–3,57^. In addition, due to unique physiological and behavioral responses at different levels of hypoxia^25,26^, gene expression associated with chronic Mild and Moderate DO should be investigated to understand organismal response. While it has been suggested that amphinomids may thrive in lowered DO conditions ^58–60^, no studies have yet determined direct gene expression responses to this stressor.

Previous work has focused on morphological changes and oxygen uptake in *H. carunculata* in response to hypoxia^27,38,39^. Most notably, branchial surface area increases in response to both intermittent^38^ and chronic^39^ hypoxia. While the effect on oxygen uptake rates during hypoxia is small^27,38^, hypoxia exposure results in a long-term elevation of the metabolic rate, potentially leading to increased feeding activity^27^. Differential gene expression will help detect additional physiological processes that affect overall organismal functioning and find explanations for the environmental hardiness of *H. carunculata*. The objectives of this study were to characterize gene expression responses in *H. carunculata* (1) under chronic Mild and Moderate hypoxia (as defined above) and (2) under severe intermittent hypoxia. Under chronic hypoxia, we hypothesize that hypoxic response genes (e.g. HIF1-α, ARNT, VHL) and metabolic genes associated with glycolysis will be up-regulated as it is an anaerobic process^32,48^. Given documented physiological responses to chronic vs intermittent hypoxia, we can expect differences in gene expression patterns as well especially with regards to oxidative stress^61,62^. When organisms undergo intermittent hypoxia, they must be able to handle times of low oxygen and high oxygen when the reactive oxygen species increase which threaten cell health and structure which can result from the formation of reactive oxygen species under hypoxia or upon reoxygenation^61,62^. For intermittent hypoxia, we predict an up-regulation of aerobic metabolism genes after normoxic timepoints and behavioral response genes (such as TRP1A) as in *Capitella teleta*^17^ after hypoxic timepoints.

## Methods

### Experimental animals

Bearded fireworms were collected in October of 2016 and November 2017, respectively, from Riviera Beach, Florida (26.783703, − 80.044643). The animals were kept at their native environmental conditions (35 ppt, 20–23 °C, and 6.80–7.25 mg O_2_ L^−1^) in the Sea Life Facility (SLF) at Texas A&M University at Galveston prior to the experiments. They were fed 0.5 g of *Loligo* spp. (squid) or penaeid shrimp by hand once per week and their consumption was observed. No significant difference in weights was observed between treatments for the chronic (2.7–8.2 g) or intermittent (2.3–12 g) trials. All worms survived the experimental trials. We additionally used 2 samples from Praia do Forte, Bahia, Brazil (− 12.559757, − 37.991274) and 1 from the Florida location (26.783703, − 80.044643) to assemble the super-transcriptome. These field-collected samples consisted of the amputated posterior ends (~ 20 segments) preserved in RNAlater and kept refrigerated until they could be transferred to a − 80 °C freezer.

### Hypoxia trials

**Figure 2:**
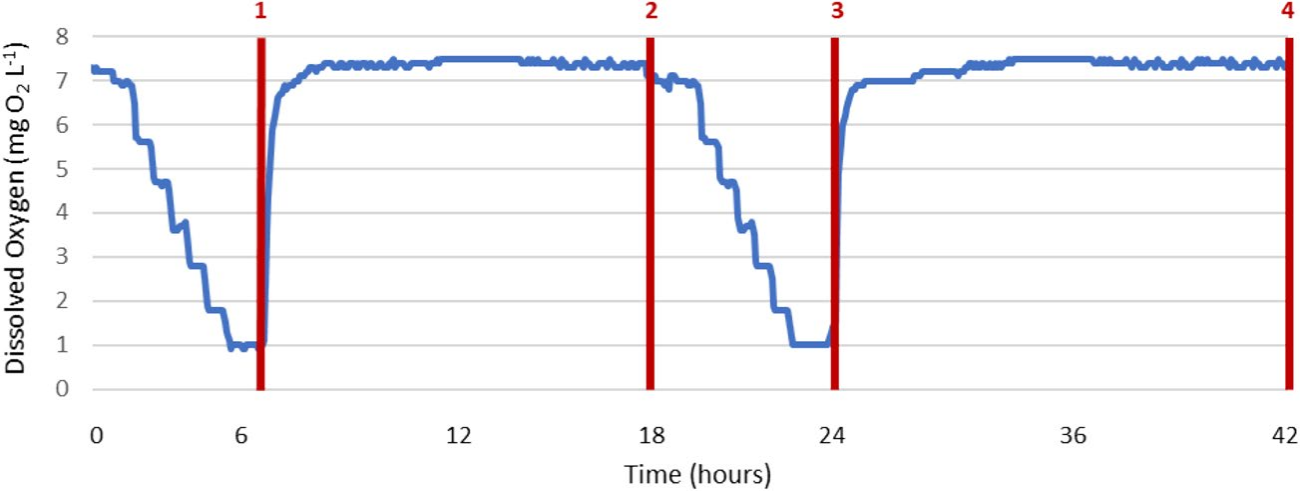
Sampling timepoints for the intermittent hypoxia trials.

Throughout the trials, the worms were not fed to reduce ammonia accumulation and decomposition. DO levels were controlled by the influx of nitrogen gas (Radnor nitrogen from Airgas Welding Supplies, TX, USA) and controlled with Neptune System’s Apex Jr. Controller. See ^33^ for more detailed descriptions and a diagram of the experimental setup. For the chronic hypoxia exposure, the DO was dropped over 48 h to desired levels for Mild and Moderate DO conditions (4.5 ± 0.25 mg O_2_ L^−1^ [64% of normoxia level] and 2.5 ± 0.25 mg O_2_ L^−1^ [36% of normoxia level], respectively). The temperature in the aquaria tanks were maintained at 20.1 ± 1.5 °C. Normoxic conditions were monitored and held steady (7.0 ± 0.25 mg O_2_ L^−1^) with a YSI Model 550A. Fireworms (n = 3 for each treatment) were sampled at the end of the 7-day trials by amputation of the posterior ~ 20 segments which were stored in RNAlater for transcriptome sequencing as described below. For the intermittent hypoxia exposure, the DO was dropped 1 mg O_2_ L^−1^ per 45 min intervals until severe hypoxia (1 mg O_2_ L^−1^ or 14% of normoxia level) was reached (Fig. 2). This DO level was then held stable for 6 h to mimic DO levels in tide pools. Fireworms (n = 3) were sampled at each time point (time point 1: 6 h [hypoxic]; time point 2: 18 h [normoxic]; time point 3: 24 h [hypoxic]; time point 4: 42 h [normoxic]) then flash frozen for later extractions and sequencing.

### RNA isolation and RNA-seq

The posterior ends from the chronic hypoxia exposure and field-collected samples were stored in RNAlater in − 80 °C until further analysis. For total RNA extraction, the tissues (n = 3 per treatment and timepoint) were homogenized with the TissueRuptor II, followed by extraction with the Qiagen RNeasy kit (Qiagen: Catalog no 74104). Tissues from the intermittent hypoxia experiments were flash frozen. For all samples, RNA was extracted from the entire homogenized fragment, without regard for potential differences among different tissue types. Total RNA was extracted using the TRIzol protocol (Life Technologies: Cat No. 15596026), followed by the Qiagen RNeasy clean up. Total RNA was quantified and quality-checked with Qubit RNA High Sensitivity (HS) Assay and SYBR Gold Nucleic Acid Gel Stain, respectively. Illumina TruSeq RNA double-stranded libraries were prepared and sequenced at Texas A&M University’s Agrilife Research Center on the Illumina NovaSeq 6000 (College Station, Texas) with paired end- 150 bp quality controlled with Fragment Analyzer through PROSize 2.0.

### Transcriptome assembly, annotation, and gene-level estimates

To account for all of the possible transcripts that could have been expressed under varying levels of DO, we assembled and annotated a “super” transcriptome with the sequences (N = 11) from the Florida collections, Brazilian collections, and the chronic hypoxia studies with default Trinity^63^ parameters and following the Trinotate pipeline (version 3.0.2)^64^, respectively. Proteins were predicted with TransDecoder (version 3.0.1) using default parameters^65^ and compared to Swiss-Prot database using NCBI BLAST + (version 2.7.1). BLASTX were conducted on contigs of the super transcriptome against non-redundant nucleotide database from UniProt (downloaded 17 June 2017). The sequences were pseudoaligned with kallisto (version 0.46.1) which identifies k-mer (given set of nucleotides) compatibility between the samples (instead of true nucleotide alignment) compared to the reference transcriptome which has been used to classify transcripts from k-mer associations in the de Bruijn graph^66^. Then, kallisto produces transcript counts for each sample which were then converted to gene counts with tximport (version 1.10.1)^67^ and analyzed for differential gene expression with edgeR (version 3.24.3)^68^. We used the TMM (trimmed-mean of M values) method for normalization of reads and CPM (counts per million) filter based on library sizes according to the edgeR protocol^68^. We also analyzed the transcripts abundances in edgeR (according to the same protocol) for comparison of genes with transcripts due to the issues with detecting true isoforms from chimeras and gene fragments when utilizing a reference transcriptome instead of a reference genome. Genes and transcripts were analyzed for differential expression with the Generalized Linear Model statistical test which is robust to low replicate numbers. Log fold change (FC) values ≥ |1| were identified as differentially expressed features (DEFs) (genes [DEGs] and transcripts [DETs]) with a false discovery rate (FDR) *p* value < 0.05. The intermittent hypoxia samples were analyzed for time course expression analysis at 4 timepoints (time point 1: 6 h [hypoxic]; time point 2: 18 h [normoxic]; time point 3: 24 h [hypoxic]; time point 4: 42 h [normoxic]) using maSigPro^69–71^ which clusters differentially expressed transcripts/genes into 9 groups that have similar expression profiles across the time points. This program uses a two-step regression analysis to (1) select differentially expressed genes with a global model then (2) identify statistically significant differences in expression between treatments (*p* < 0.05 and R > 0.7)^69^. Data available on FigShare at https://figshare.com/s/700e408ff5beae60225e.

## Results

**Figure 3:**
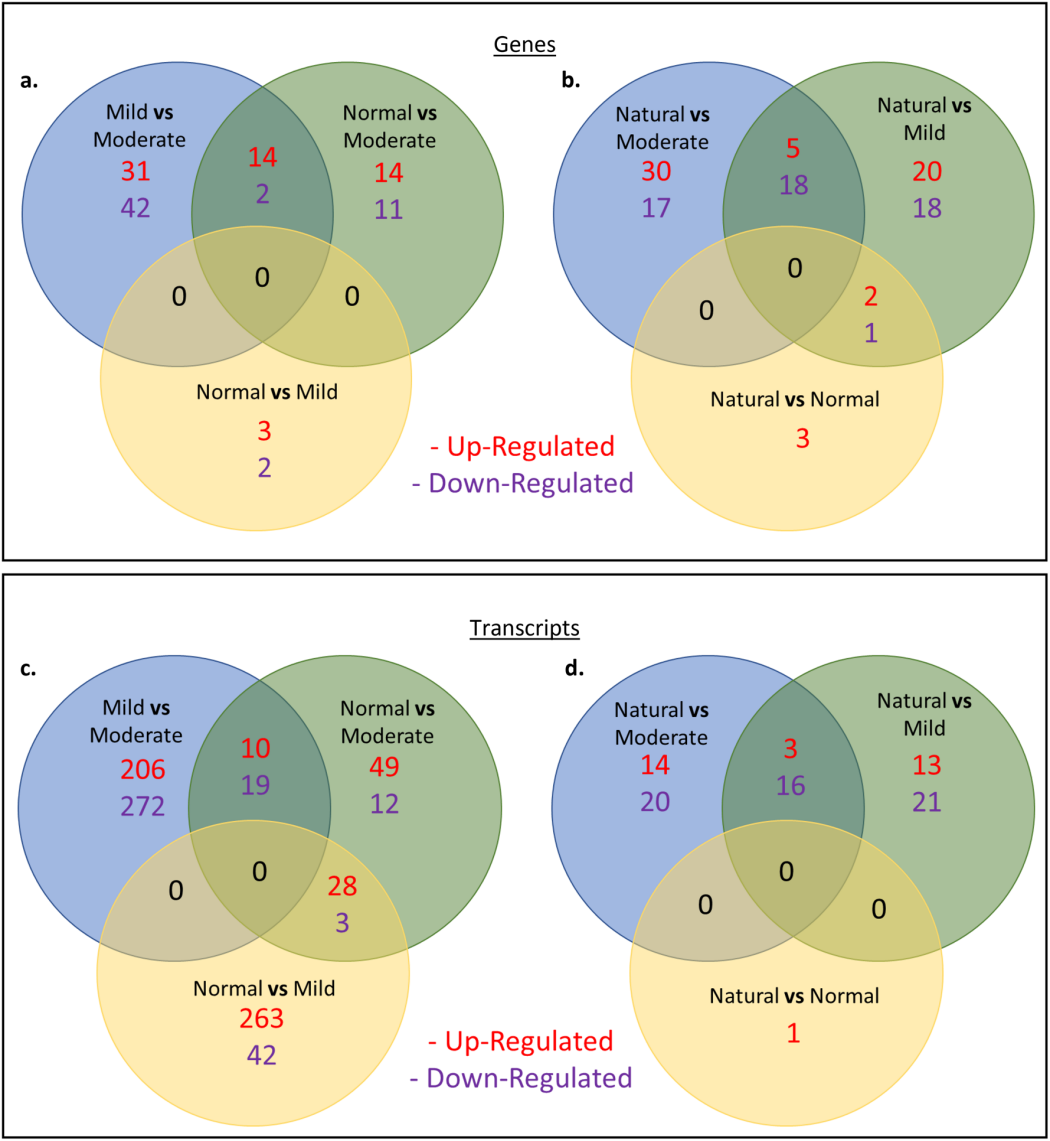
Differentially expressed genes (**a**, **b**) and transcripts (**c**, **d**) from chronic hypoxia treatments. On the left, we compared the chronic hypoxia treatments (**a**, **c**), and on the right, (**b**, **d**) those worms in captivity for the chronic hypoxia trial were compared to those sampled from the natural environment (Brazil and Florida).

Preliminary investigation of *H. carunculata’s* transcriptome revealed homologs necessary for hypoxic response (such as HIF-1α, ARNT, VHL, TRP1A, PHD, etc.). The super transcriptome was analyzed for estimated completeness (94.7%) with a BUSCO analysis (Eukarya—S: 61.7%, D: 33.0%, F: 5.3%, M: 0.0%, n: 303). The number of total Trinity transcripts and subsequent genes are shown in Table 1 from 23 samples of *H. carunculata* with 3 biological replicates for per treatment (except Normal during chronic hypoxia = 2). Contigs with BLAST hits corresponding to Viruses, Bacteria, or Archaea were removed from this analysis. During chronic hypoxia, 122 DEGs and 587 DETs were down-regulated while 112 DEGs and 405 DETs were up-regulated (Fig. 3a–d).

**Table 1:** Transcript and gene counts from hypoxia trials.

Experiment	Treatment	Avg transcript no	Transcript Std	Avg gene no	Gene Std
Intermittent	Hypoxia	12,986,386	2,137,850	4,427,765	1,038,293
	Normoxia	16,111,798	1,807,594	2,869,847	677,219
	Hypoxia	15,051,339	1,423,484	4,337,749	486,333
	Normoxia	17,398,457	1,872,418	4,200,182	589,610
Chronic	Normoxia	2,225,688	1,085,822	1,075,336	665,078
	Mild	4,559,582	833,144	758,377	113,415
	Moderate	3,306,403	932,714	512,957	197,699
	Natural	4,103,627	3,131,778	1,027,738	275,583

Std = standard deviation.

### Chronic hypoxia trials

The highest number of DEGs (89 genes) occurred in the Mild vs. Moderate comparison, followed by Normal vs Moderate (32 genes). A unique set of 5 genes were differentially expressed in the Normal vs. Mild comparison (Fig. 3a). When comparing fireworms from the lab to field-collected worms, the number of DEGs progressed with increasing levels of hypoxia (Normal [6] <  Mild [64] <  Moderate [70]) (Fig. 3b). The DEFs between organisms in laboratory trials and those from the field largely consisted of metabolism-associated features and unclassified sequences.

Anaerobic metabolism genes associated with glycolysis were up-regulated in the chronic hypoxia treatments (Tables 2, 3) as opposed to those associated with cell adhesion, transport, and development which were down-regulated. There was also a significant up-regulation of HIF-1α in the Mild DO treatment compared to Normal and Moderate DO treatments, and an up-regulation of ARNT in the Moderate DO treatment compared to Normal (Tables 3, 4, Fig. 5). Heme-binding protein (CP3AD) was down-regulated in the Moderate treatment while oxygen-binding (extracellular hemoglobin) was up-regulated. However, there was a decrease in the expression of metabolic genes and an increase in those associated with cell differentiation, digestive tract, and reproductive system development.

**Table 2:** Selected down-regulated features under chronic lowered oxygen conditions in the bearded fireworm, Hermodice carunculata, based on pairwise comparisons.

Description	Treatment	UniProt	Log FC	Homolog name
Metabolism	Moderate	DYM	− 8.57	Dymeclin
	Moderate	DHA	− 8.85	Alanine dehydrogenase
	Mild	ATP6	− 7.05	ATP-dependent RNA-helicase
	Mild	COX1	− 8.18	Cytochrome c oxidase subunit 1
	Mild	CYB	− 6.10	Cytochrome-b
	Mild	ASMT	− 8.59	Acetylserotonin O-methyltransferase
	Mild	S22AI	− 8.20	Solute-carrier family 22
	Mild	RBM39	− 8.67	RNA-binding protein
	Mild	RADI	− 8.47	Radixin
Response to stress	Mild	RAC1	− 9.10	Ras-related C3 botulinum toxin
Transport	Mild	DX39A	− 9.67	Transport protein
	Mild, moderate	SC61B	− 6.69, − 9.28	Protein transport protein
Development	Mild	TEKT3	− 9.73	Tektin
	Mild	LRP4	− 9.41	Low density lipoprotein
	Mild	NOTC1	− 8.61	Neurogenic locus notch 1
	Moderate	CO1A2	− 5.41	Collagen-alpha-2 (1)
Translation	Mild, moderate	RL13A	− 4.59, − 5.29	60 s ribosomal protein L13
	Mild	RL6	− 5.64	60 s ribosomal protein
Cell adhesion	Moderate	CSPG2	− 7.42	Versican core protein

**Table 3:** Selected up-regulated features under chronic hypoxic conditions.

Description	Treatment	UniProt	Log FC	Homolog name
Metabolism	Mild,Moderate	TENS1	9.85, 9.53	Tensin-1
	Mild,Moderate	RL14	6.61, 6.56	60 s ribosomal protein
	Mild,Moderate	ACTN	10.13, 11.39	Alpha-actin
	Mild,Moderate	HAAF	9.44, 8.96	Hemagluttinin/amebocyte aggregation
	Mild,Moderate	FACR1	9.27, 9.14	Fatty-acyl CoA reductase
	Mild	CANB	8.93	Calpain-B
	Mild, Moderate	GEPRP	9.11, 9.44	Glycine-glutamate rich protein
	Moderate	FLNA	9.56	Filamin A
Development	Mild,Moderate	BOLL	9.51, 9.14	Protein boule-like
	Mild,Moderate	CSMD3	8.58, 9.12	Cub and sushi domain
	Mild,Moderate	USOM7	5.58, 5.91	Uncharacterized skeletal protein
	Mild,Moderate	FACR1	9.27, 9.14	Fatty-acyl CoA reductase
	Mild	NRG	10.54	Neuroglian
	Mild	NDST	9.38	Bifunctional heparan
Transcription	Mild,Moderate	DDX21	8.68, 8.97	Nucleolar RNA-helicase
	Mild	CRERF	9.08	Protein CREBRF
	Mild	MRTFB	9.10	Myocardin-related transcription factor
Locomotion	Mild	UNC22	3.85	Twitchin
Chitin-binding	Mild	CPG2	4.20	Chondroitin
Heme-binding	Mild	CP1A1	4.86	Cytochrome P450 1A1
Response to hypoxia	Moderate	ARNT	8.06	Aryl-hydrocarbon receptor nuclear translocator
	Mild	HIF1A	3.63	Hypoxia inducible factor 1-alpha

**Table 4:** Selected up-and down-regulated genes in Moderate DO treatment compared to Mild DO treatment.

Regulation	Description	GenBank	Log FC	Homolog name
Down	Metabolism	NU2M	− 8.39	NADH-ubiquinone oxidoreductase 2
		ATP6	− 5.10	ATP synthase subunit a
		COX2	− 2.05	Cytochrome c oxidase subunit 2
		GNAO	− 8.78	Guanine nucleotide-binding
	Response to hypoxia	HIF1A	− 8.71	Hypoxia inducible factor 1-alpha
	Heme-binding	CP3AD	− 9.22	Cytochrome P450 3A13
Up	Metabolism	NU5M	8.55	NADH-ubiquinone oxidoreductase 5
		KINH	8.19	Kinesin Heavy Chain
		MLP	7.80	Mucin-like
	Development	NOTC1	7.79	Neurogenic locus notch 1
		ATC1	7.66	Calcium-transporting ATPase
		PTP10	6.17	Tyrosine-protein phosphatase
	Oxygen-binding	GLBB2	8.36	Extracellular giant hemoglobin
	Transport	MOT10	7.45	Monocarboxylate transporter

### Intermittent hypoxia trials

**Figure 4:**
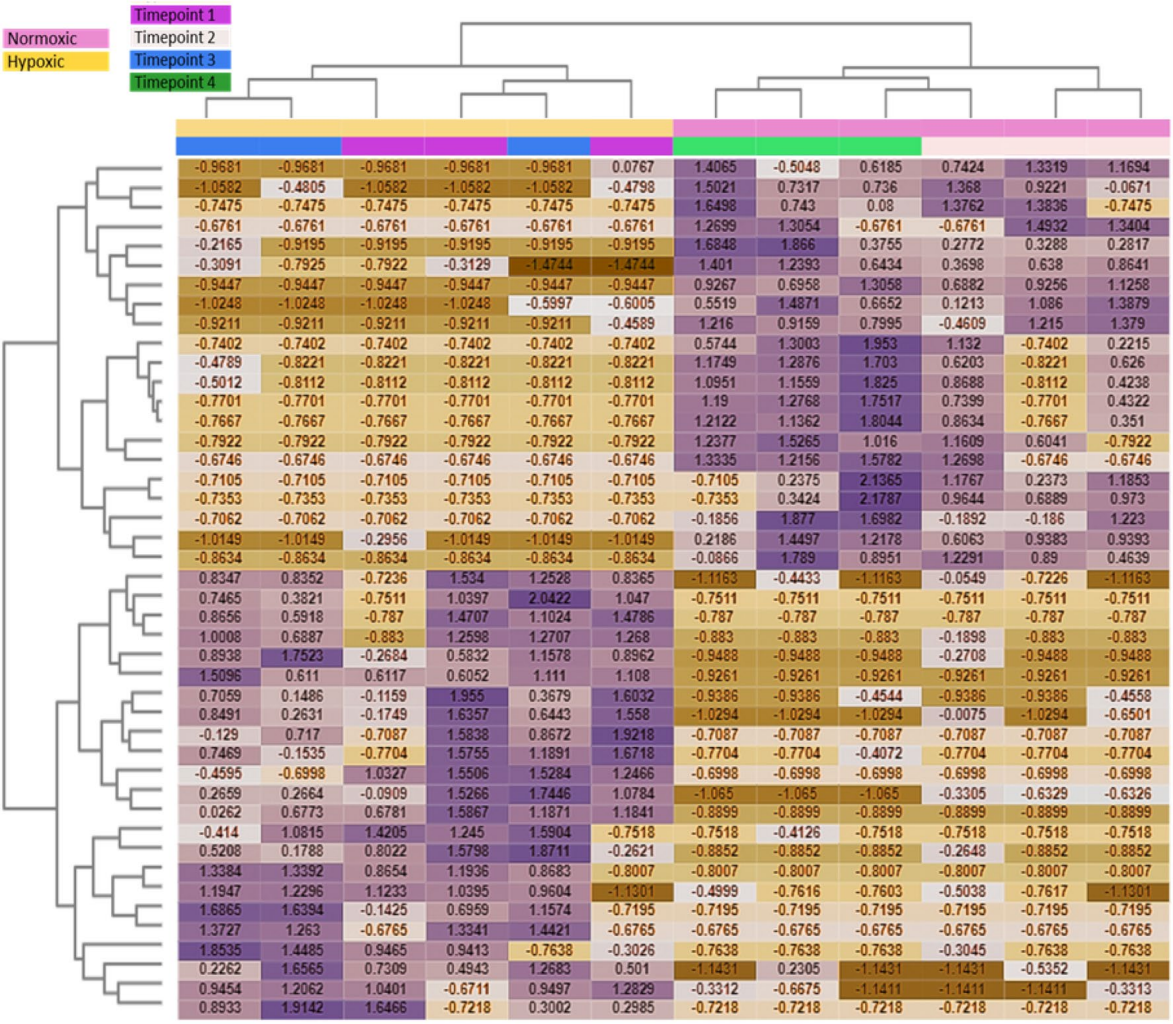
Heatmap of the top 44 differentially expressed genes for each sample after the intermittent hypoxia trial with the treatments and timepoints. Values within the heatmap are counts per million (CPM) that that have been selected as differentially expressed based on log fold-change (FC) values with a ± 1 cutoff. Time point 1: 6 h (hypoxic); time point 2: 18 h (normoxic); time point 3: 24 h (hypoxic); time point 4: 42 h (normoxic).

The inclusion of timepoints in the pairwise differential expression resulted in 0 DEGs and 65 DETs up-regulated compared with 3 DEGs and 67 DETs down-regulated (Fig. 4). There was a distinction between hypoxia and normoxia conditions, but timepoints within treatments were not separated in the dendrogram (Fig. 4, top). The majority of DEFs between conditions serve metabolic functions, nucleotide binding, GTPase, and polymerase activity. However, heme-transporter activity (proton-coupled folate transporter) was down-regulated at the hypoxic timepoints and protein ubiquitination was down-regulated at the normoxic timepoints.

Our timepoint analysis for intermittent hypoxia revealed 9 DEF clusters with characteristic expression profiles (Table 5). In clusters 3, 4, and 7, expression patterns followed similar trends for the normoxic and hypoxic timepoints (line trends in Table 5); however, DEFs in cluster 4 had higher expression levels under normoxia, whereas DEFs in clusters 3 and 7 were more expressed under normoxia. Cluster 1 contained genes associated with epithelial cell morphogenesis and metabolism and were expressed at higher levels after the first timepoint of hypoxia but decreased to around normal levels after the second 6-h hypoxia block (timepoint 3). Cluster 2 genes, consisting primarily of heme-binding, metabolism, and translation features, increased after both hypoxic and normoxic timepoints; however, the increase at hypoxia timepoints was more drastic. Genes in cluster 3 were expressed at higher levels in hypoxic timepoints consisting of increased heme-binding affinity and increased production of red blood cells. We did not see any differential expression of HIFs in intermittent hypoxia; however, we did see an initial decrease in expression of Usp19 (Ubiquitin carboxyl-terminal hydrolase 19) after hypoxia timepoint 1 compared to normoxia timepoint 2 but equally expressed after timepoints 3 and 4 (Cluster 5). Genes associated with embryonic development, cell proliferation, and angiogenesis were up-regulated during hypoxia (Cluster 7). The largest slope increase and decrease in expression trends occurred in cluster 8 for hypoxic and normoxic timepoints, respectively. The large positive slope associated with cluster 8 under hypoxia, nearly doubles in expression between to the two timepoints. Cluster 8 genes had inverse expression trends between hypoxic and normoxic timepoints and contained genes associated with epidermal growth factor-, actin-, and Notch-binding.

**Table 5:** Selected (4) top hits based on percent identity and e-value for time course cluster analysis from intermittent hypoxia trials with expression trends shown over the 42 h of the experiment (from left to right).

Cluster	UniProt hit	Homolog name	Function
1 280 features	HAAF	Hemagglutinin/amoebocyte aggregation	Trigger aggregation of amebocytes and erythrocytes
	FRAS1	Extracellular matrix protein fras1	Morphogenesis of epithelium
	NU5M	NADH-ubiquinone oxidoreductase 5	Metabolism
	HSP7C	Heat shock cognate 71	Protein chaperone in many processes
2	DDX18	ATP-dependent RNA helicase	Metabolism
86 features	CP4F1	Cytochrome P450 4F1	Heme-binding
	RL13A	60 s Ribosomal protein L13a	Translation
	MEGF6	Multiple epidermal growth factor	Calcium ion binding
3	CP270	Cytochrome P450 C270	Heme-binding
205 features	NTCP2	Ileal sodium cotransporter	Sodium ion transport
	CALM	Calmodulin	Calcium-mediated signaling
	HPGDS	Hematopoeitic prostaglandin D synthase	Protein homodimerization activity
4 157 features	POSTN	Periostin	Heparin-binding and cell adhesion
	NRG	Neuroglian	Developmental protein
	SLO1	Calcium-activated potassium channel	Potassium ion transport
	PDCD6	Programmed cell death protein 6	Regulation of angiogenesis
5 258 features	FIBP	Acidic fibroblast growth factor intracellular-binding protein B	May help in laterality development
	UBP19	Ubiquitin carboxyl-terminal hydrolase 19	Hsp90 protein binding
	RFA1	Replication protein A 70 kDa DNA-binding subunit	DNA repair and telomere maintenance
	HEM6	Oxygen-dependent coproporphyrinogen-III oxidase	Heme/porphyrin biosynthesis
6 267 features	OTOP2	Otoperin	Hydrogen ion transport
	PRDX1	Peroxiredoxin-1	Cell protection against oxidative stress
	SQSTM	Sequestosome-1	Ubiquitin-binding
	GEPRP	Glycine, glutamate and proline-rich protein	Peptidoglycan catabolic process
7 149 features	FOLR1	Folate receptor alpha	Embryonic development and cell proliferation
	MLRA	Myosin regulatory chain A	Muscle protein
	FLNA	Filamin-A	Angiogenesis/blood vessel remodeling
	PON2	Arylesterase 2	Response to oxidative stress
	KCRT	Creatine kinase	ATP-binding
8	TSP4	Thrombospondin-4	Cell adhesion and growth factor
110 features	TENS1	Tensin-1	Actin-binding
	S40A1	Solute carrier family 40 member 1	Iron ion transmembrane transporter
	FBP1	Fibropellin-1	NOTCH binding
9 189 features	U520	Putative U5 small nuclear ribonucleoprotein 200 kDa helicase	ATP/RNA-binding
	HSP71	Heat shock 70 protein 1	Protein folding chaperone
	UBIQP	Polyubiquitin	Different functions depending on what protein it is attached to
	CP1A1	Cytochrome p450 1A1	Heme-binding

Expression profiles are shown under the cluster number: full lines are hypoxic time points and dashed lines are normoxic time points. For example, expression of the genes in cluster 1, both hypoxic and normoxic, declined over the course of the experiment, but hypoxic expression declined at a faster rate (steeper slope).

## Discussion

Our chronic trials revealed down-regulation of genes related to cell adhesion, transport, development and heme-binding, and up-regulation of genes related to glycolysis, oxygen binding, cell differentiation, digestive, and reproductive function under hypoxic conditions. The overall up-regulation of DEFs associated with metabolic functions in the Moderate DO fireworms indicates a stress response through shifted metabolism in response to chronic hypoxia from aerobic to anaerobic (Tables 2, 3). We found differential expression of metabolic genes between Mild and Moderate treatments indicating modulated responses (e.g. down-regulation of aerobic metabolic pathways to hypoxic exposure based on level of severity) in Table 4. For example, we saw a down-regulation of ATP synthase and cytochrome c oxidase, key members of the electron transport (Table 4). This response has been shown in several invertebrate species and shows that fireworms have a similar physiological response to hypoxia as the Pacific oyster, *Crassostrea gigas*, and the water flea, *Daphnia magna*^42,48,51^.

**Figure 5:**
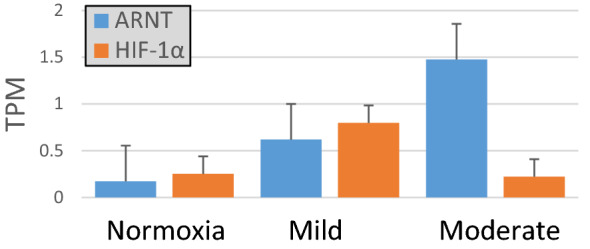
Transcripts per million (TPM) expression values for the two subunits of the HIF transcription factor during chronic hypoxia.

The two subunits of the HIF transcription factor, HIF-1α and ARNT, are constitutively expressed, even under normoxic conditions, but a functional transcription factor only forms under hypoxia when the oxygen-dependent degradation of HIF-1α is disrupted. Once dimerized, HIF triggers downstream hypoxia responses in the organism^21,30–32^. However, few studies have examined the differential expression of the two HIF subunits in response to different levels of hypoxia. We found that both HIF-1α and ARNT are upregulated under Mild hypoxia (Fig. 5), although the upregulation was only significant for HIF-1α. Under Moderate hypoxia, only ARNT is significantly upregulated whereas HIF-1α expression decreases. The increase in ARNT expression from Normal to Mild to Moderate chronic hypoxia (Fig. 5) further indicates a modulated response to chronic hypoxia exposure dependent upon DO level. This implies that downstream hypoxia responses may not only be triggered through oxygen-dependent degradation of HIF-1α, but possibly also through a modulation of expression levels depending on DO levels. The higher expression of HIF-1α after Mild chronic hypoxia but return to normal expression at moderate levels could indicate yet unknown underlying acclimatory responses to this chronic condition.

As in *Paralvinella* sp.^52,53^, extracellular hemoglobin was up-regulated in the fireworms after Moderate chronic hypoxia compared to Mild hypoxia (Table 4). Another invertebrate blood pigment, hemocyanin, has putatively been found in the transcriptomes of a closely related fireworm, *Paramphinome jeffreysii*^72^, and was down-regulated in the bearded fireworm after Moderate chronic hypoxia compared to normoxic conditions. The increased production of extracellular hemoglobin and decreased production of hemocyanin in Moderate hypoxia suggests that the two pigments are optimized for different DO levels in the blood; however, a more targeted study is necessary to understand this relationship.

Our control DO levels were similar to prevailing conditions around the time of capture of the field-collected specimens; consequently, the fewest DEFs were found between the Normal treatment and the field-collected worms. Also, the down-regulation of certain metabolic genes associated with aerobic metabolism when comparing the animals exposed to lowered DO conditions to those of their natural habitat indicates the overall metabolic depression of worms exposed to chronic hypoxia. These results therefore support a previous study demonstrating lowered oxygen uptake in chronic hypoxic fireworms^27^.

When analyzing the top DEFs during the intermittent hypoxia trials, no clear separation of timepoints within the hypoxic treatment was obvious, but there was a distinction between the normoxic timepoints (Fig. 4, top left and right, respectively). While most of the differentially expressed genes in the Fig. 4 remained unclassified, a few in the upper right section (expressed more after normoxic timepoints) were putatively characterized as genes related to aerobic metabolism and cell adhesion, such as Cytochrome c oxidase subunit 1 (COX1) and periostin. However, the time course cluster analysis revealed some expression trends depending on time points (Table 5). The worms used in this study had likely never been exposed to hypoxia before, so it is not surprising that we see different expression trends at timepoint 1 and 3. The increase in expression of genes related to epithelial morphogenesis after 6 h (Table 5) may be the first sign of increasing branchial filament formation in this species to cope with hypoxic conditions, which occurs both under intermittent^38^ and chronic hypoxia^39^. Although fireworms most likely cannot increase their branchial surface area within six hours, this short time may be sufficient to trigger the onset of branchial tissue formation.

The consistent higher expression of hematopoietic prostaglandin D synthase (Cluster 3) after the hypoxic timepoints suggests physiological responses related to vasodilation and inhibition of clotting so that blood transporting oxygen can flow more freely^73^. This short-term response to intermittent hypoxic stress may increase blood flow and oxygen transport to maintain necessary metabolic functions during times of lowered oxygen conditions.

Ubiquitin-specific protease 19 (Usp19) protects HIF-1α from degradation and is required for typical hypoxia response in vertebrates^74,75^. If Usp19 is absent under oxygen depletion, HIF-1α will follow an oxygen-independent pathway to degradation not shown in Fig. 1a^75,76^. Usp19 interacts with the N-terminus of HIF-1α which is also where the chaperone, Heat shock protein 90 (Hsp90), associates to stabilize HIF-1α (Fig. 1a). In our experiment, Usp19 was initially less expressed after hypoxic timepoint 1 compared to normoxia (Cluster 5 in Table 5), possibly indicating the destabilization of the HIF-1α chaperone which may allow it to dimerize with ARNT more rapidly. However, Usp19’s similar expression level at timepoints 3 and 4 suggests it has other roles under normoxic conditions as well. Typically, under hypoxic conditions, HIF-1α is protected from proteasomal degradation by Usp19, but the expression trends (higher expression after first hypoxic exposure) here suggest there may be another protective mechanism for HIF-1α in the fireworm and potentially other animals.

Upon comparison of the intermittent and chronic studies, we found consistent regulation of a TRP1A homolog across all hypoxia and normoxic conditions and timepoints supporting its role in oxygen sensing in annelids^17^. Heme-binding proteins were more highly expressed after the hypoxia timepoints during intermittent hypoxia and portrayed a positive slope as time progressed indicating increased expression after repeated hypoxic exposure. Compared with chronic hypoxia, heme-binding proteins were up-regulated after Mild hypoxic exposure compared with Normal, followed by a down-regulation in severe compared with Mild. Such a result may entail several stages of hypoxic response in this species depending on DO level, exposure time, and frequency of exposure. It may be necessary to reach a certain level of lowered DO (4.5 mg O_2_ L^−1^) before heme-binding protein genes are up-regulated in response to hypoxia, but then they may be down-regulated if the production of the protein is too energetically expensive. However, more research would need to be done to further support this suggestion.

Filamin A (FLNA) is a structural protein associated with actin-binding that has been described as important for cancer tumor growth in the lungs^77^, and we saw it up-regulated in the Moderate chronic hypoxia treatment compared to Normal. In addition, in the intermittent trials, FLNA was consistently more expressed after hypoxic timepoints indicating a similar response to chronic vs intermittent hypoxia. Since this protein is involved with new growth of cancerous tumors and other cell types (such as blood vessels^78^), the higher expression after chronic Moderate and intermittent hypoxia supports the idea that lowered oxygen levels triggers growth in specific cell types.

The different expression patterns of hematopoietic prostaglandin D synthase during intermittent hypoxia, but absence during chronic hypoxia indicates that this may be purely a short-term reaction to hypoxic stress. After the initial stress, the organism seems to shift to morphological and longer-term responses (such as metabolic depression and development of new blood vessels) to cope with hypoxia. *H. carunculata* has previously been shown to exhibit plasticity in its branchial morphology to cope with hypoxic conditions^38,39,41^, but the underlying molecular mechanisms for these changes have not previously been described. Our study shows that hypoxia responses in *H. carunculata* can be modulated depending on the type (intermittent vs. chronic) and the severity of hypoxia exposure.

Our chronic hypoxia experiments showed that Notch-binding was up-regulated in the Moderate treatment compared to the Mild but was down-regulated in the Mild treatment compared to Normal indicating a differential response that is dependent on DO levels. We also saw differential expression of Notch associated proteins between the chronic and intermittent hypoxia trials. The up-regulation of Notch in Moderate hypoxia compared to Mild was similar to the drastic increase in regulation between timepoints 1 and 3 in the intermittent trials. The up-regulation of Notch proteins, associated with development (especially that of the heart) under hypoxia conditions has been described in zebrafish, *Danio rerio*^32^. Likewise, hypoxia-triggered up-regulation of Notch-binding proteins has been shown to increase tracheal formation in insects^79^. Our findings suggest that these three divergent phyla, representing the three major branches of the Bilateria (Deuterostomia, Ecdysozoa, Spiralia/Lophotrochozoa) may utilize similar signaling pathways to alter their vastly different oxygen-delivery systems. The role of Notch-binding in oxygen delivery in lophotrochozoans requires further study. In addition, Notch signaling has been shown to increase expression of HIF-1α in hypoxic conditions^80^, so its importance in development and response to hypoxia has been exemplified here again.

Fibropellin-1, related to Notch protein-binding, was recently shown to have drastically increased in expression post chronic hypoxia in the clam *Ruditapes philippinarum*^81^. In the current study, we see a similar expression pattern in the intermittent but not chronic hypoxia trial. We saw an increase in expression of Fibropellin-1 in the bearded fireworm after the second hypoxic timepoint in the intermittent hypoxia trial (over 18 h after initial hypoxic exposure) which corresponds well with the clam’s drastic increase in this gene after 2 days of chronic hypoxia. However, it is important to note that a significant decrease in expression of Fibropellin-1 was seen after 5 and 8 days in the clam^81^. During our chronic hypoxia exposure experiment, we were only able to sample after 7 days, so we may have missed this spike in expression, but our intermittent experiment allows us to see a similar response.

Although our biological replicates were limited to 2–3 individuals per treatment, our gene expression analyses discerned statistically significant differences and trends between groups exposed to different types (chronic vs. intermittent) and levels of hypoxia. Many of the gene expression responses that we observed are consistent with previous observations. For example, the higher expression levels of genes related to epithelial morphogenesis are consistent with the increase in surface branchial surface area^39^; likewise, expression patterns of metabolism-related genes correspond with our observation of oxygen uptake^27^. Other observed trends, such as the expression patterns of genes in the HIF or Notch pathways, can guide future biochemical and physiological studies in *H. carunculata* or other species.

This study showed that six hours of hypoxia exposure is sufficient to initiate molecular pathways for epithelial morphogenesis and blood vessel remodeling, providing further support that morphological changes of respiratory structures are not reliable taxonomic characters in amphinomids (or even possibly in annelids in general). Our data strongly support the notion that *H. carunculata* can swiftly initiate physiological and morphological responses to hypoxia^38,41^ even if a behavioral response (avoidance) is not an option. This study supports our previous prediction^33^ that *H. carunculata* will thrive under changing ocean conditions and may experience population increases that could potentially have damaging effects on coral reefs and other sensitive marine habitats in its distribution range.
